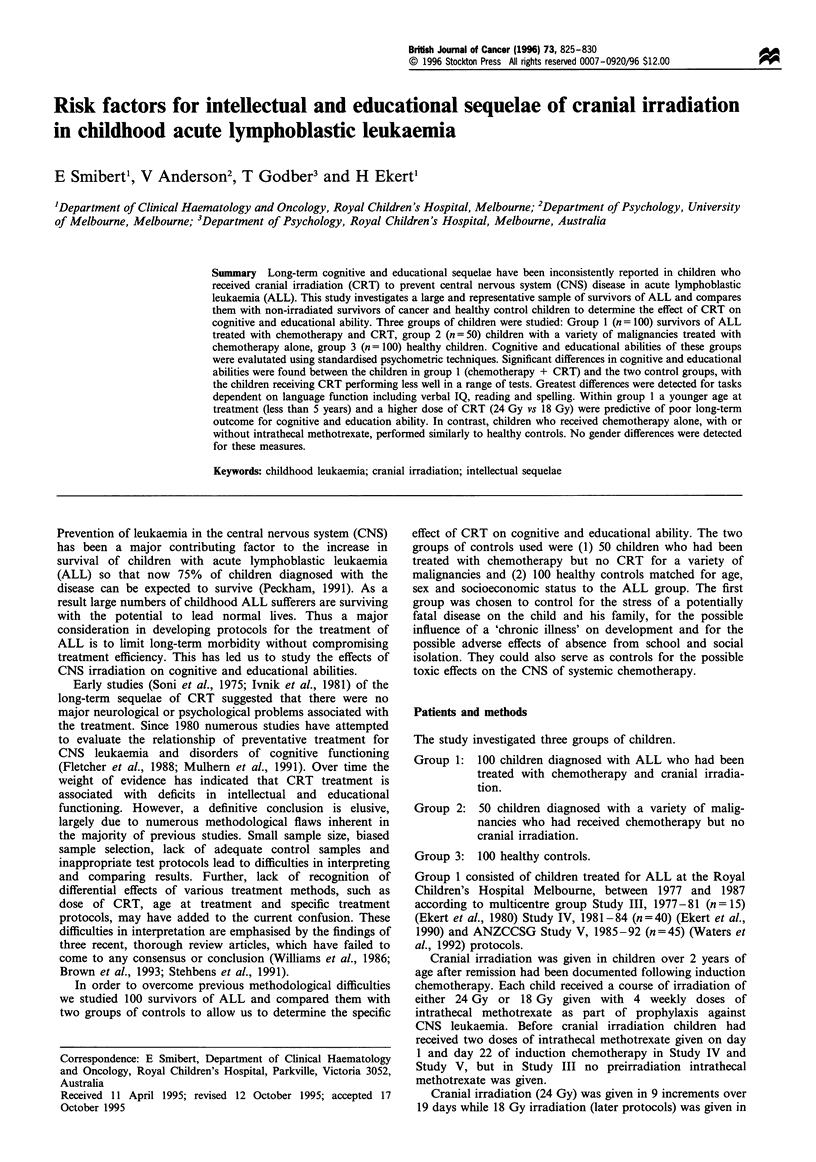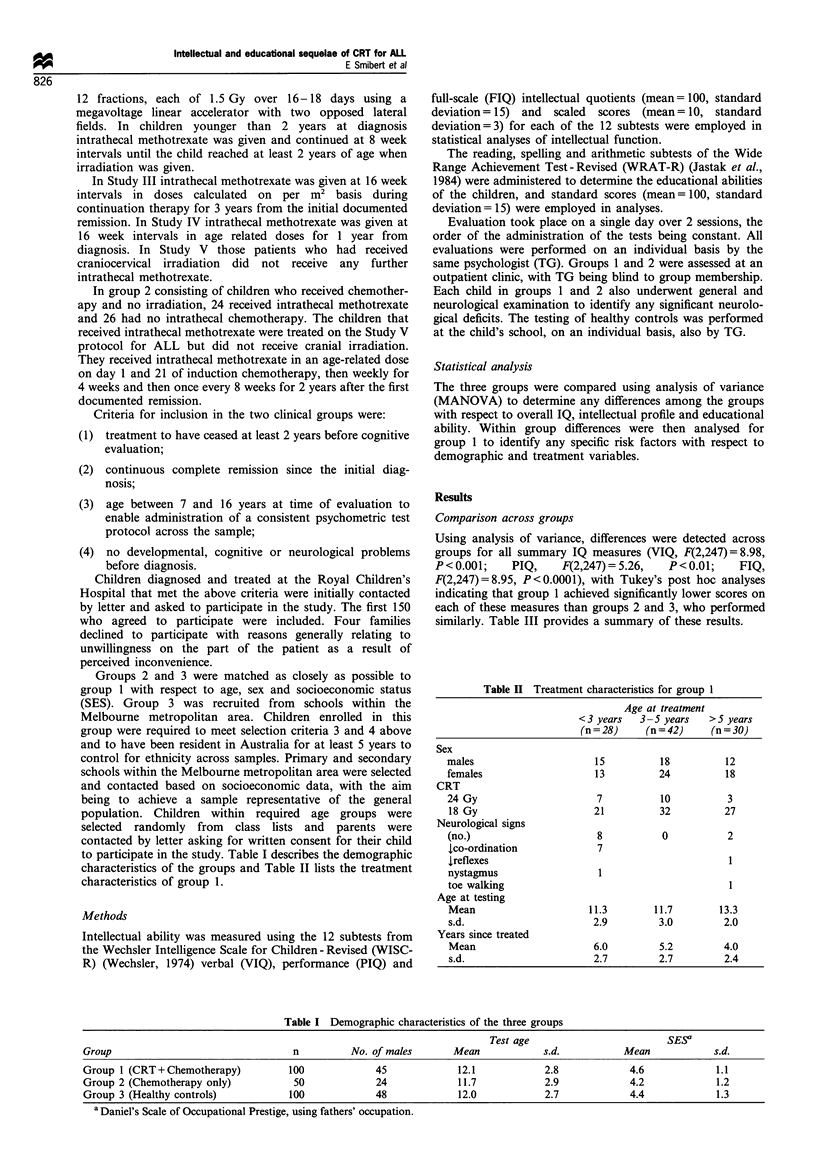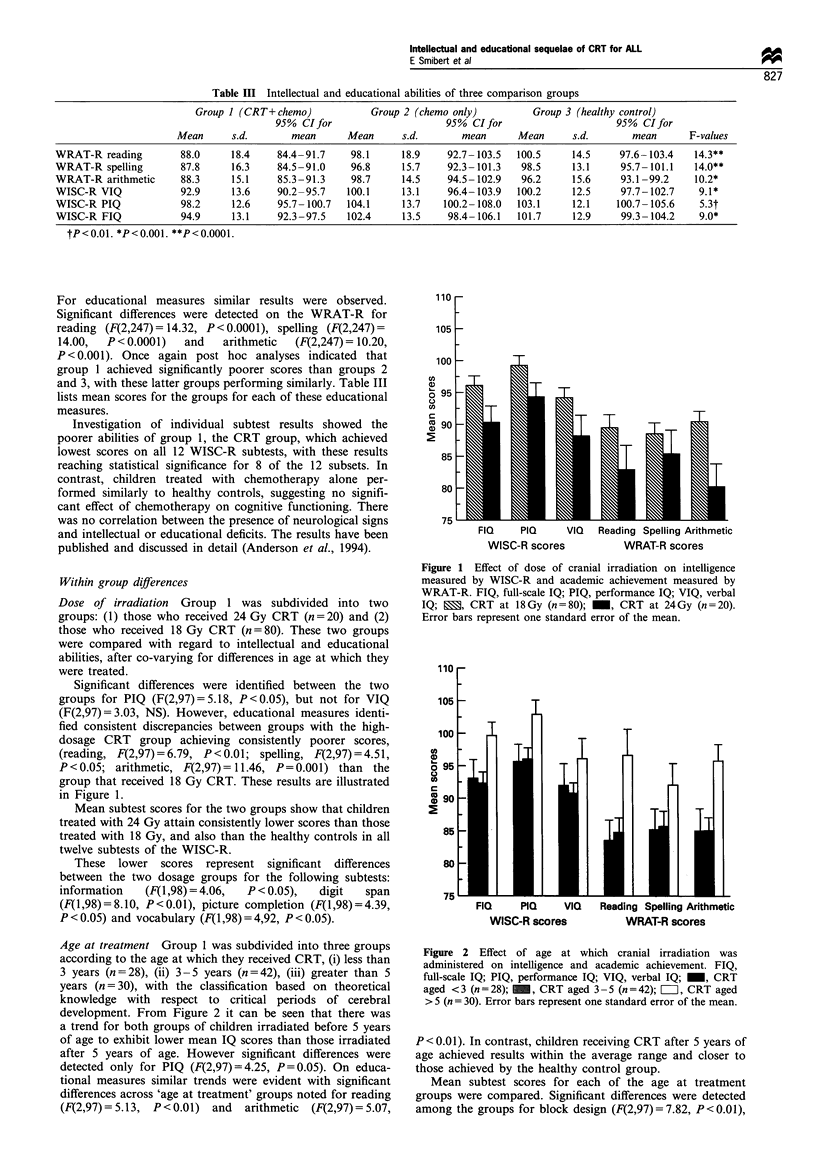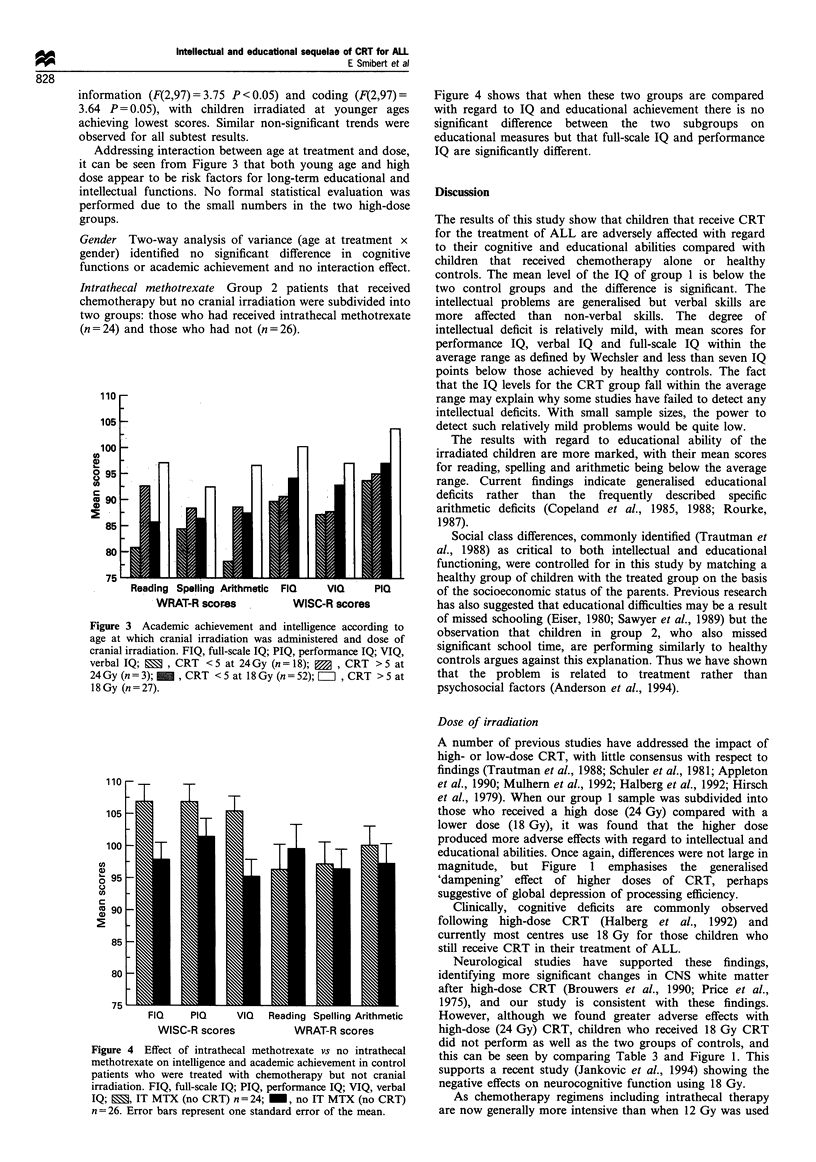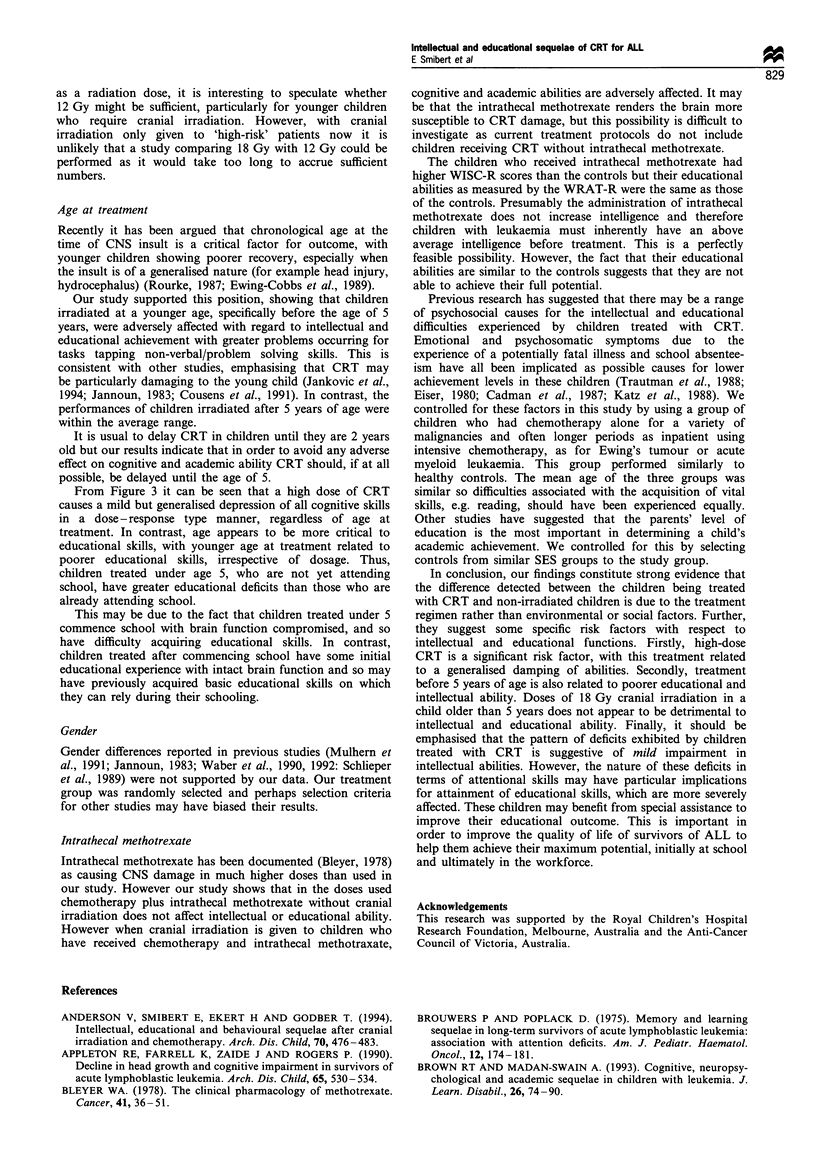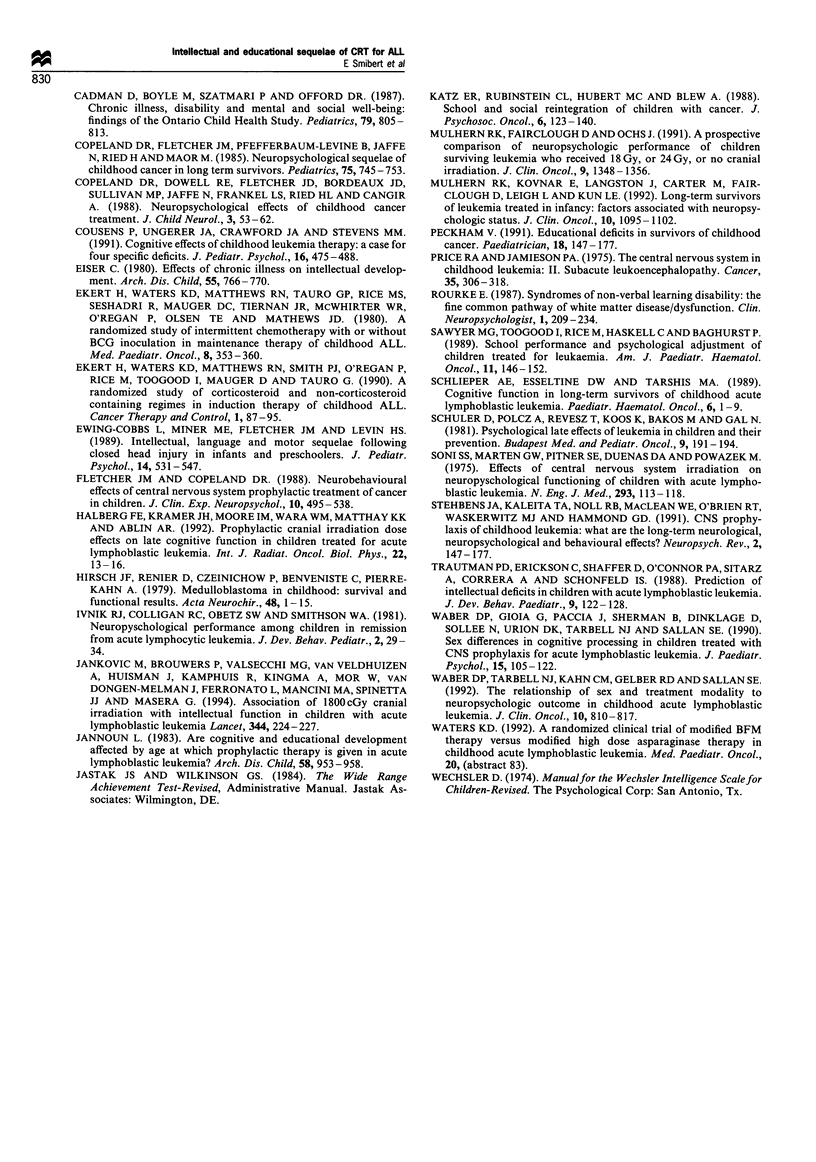# Risk factors for intellectual and educational sequelae of cranial irradiation in childhood acute lymphoblastic leukaemia.

**DOI:** 10.1038/bjc.1996.145

**Published:** 1996-03

**Authors:** E. Smibert, V. Anderson, T. Godber, H. Ekert

**Affiliations:** Department of Clinical Haematology and Oncology, Royal Children's Hospital, Melbourne, Australia.

## Abstract

Long-term cognitive and educational sequelae have been inconsistently reported in children who received cranial irradiation (CRT) to prevent central nervous system (CNS) disease in acute lymphoblastic leukaemia (ALL). This study investigates a large and representative sample of survivors of ALL and compares them with non-irradiated survivors of cancer and healthy control children to determine the effect of CRT on cognitive and educational ability. Three groups of children were studied: Group 1 (n=100) survivors of ALL treated with chemotherapy and CRT, group 2 (n=50) children with a variety of malignancies treated with chemotherapy alone, group 3(n=100) healthy children. Cognitive and educational abilities of these groups were evaluated using standardised psychometric techniques. Significant differences in cognitive and educational abilities were found between the children in group 1 (chemotherapy + CRT) and the two control groups, with the children receiving CRT performing less well in a range of tests. Greatest differences were detected for tasks dependent on language function including verbal IQ, reading and spelling. Within group 1 a younger age at treatment (less than 5 years) and a higher dose of CRT (24 Gy vs 18 Gy) were predictive of poor long-term outcome for cognitive and education ability. In contrast, children who received chemotherapy alone, with or without intrathecal methotrexate, performed similarly to healthy controls. No gender differences were detected for these measures.


British Journal of Cancer (1996) 73, 825-830
© 1996 Stockton Press All rights reserved 0007-0920/96 $12.00

### Risk factors for intellectual and educational sequelae of cranial irradiation in childhood acute lymphoblastic leukaemia

E Smibert[1], V Anderson[2], T Godber[3] and H Ekert[1]

[1]Department of Clinical Haematology and Oncology, Royal Children's Hospital, Melbourne; [2]Department of Psychology, University of Melbourne, Melbourne; [3]Department of Psychology, Royal Children's Hospital, Melbourne, Australia

**Summary** Long-term cognitive and educational sequelae have been inconsistently reported in children who received cranial irradiation (CRT) to prevent central nervous system (CNS) disease in acute lymphoblastic leukaemia (ALL). This study investigates a large and representative sample of survivors of ALL and compares them with non-irradiated survivors of cancer and healthy control children to determine the effect of CRT on cognitive and educational ability. Three groups of children were studied: Group 1 ($n=100$) survivors of ALL treated with chemotherapy and CRT, group 2 ($n=50$) children with a variety of malignancies treated with chemotherapy alone, group 3 ($n=100$) healthy children. Cognitive and educational abilities of these groups were evalutated using standardised psychometric techniques. Significant differences in cognitive and educational abilities were found between the children in group 1 (chemotherapy + CRT) and the two control groups, with the children receiving CRT performing less well in a range of tests. Greatest differences were detected for tasks dependent on language function including verbal IQ, reading and spelling. Within group 1 a younger age at treatment (less than 5 years) and a higher dose of CRT (24 Gy *vs* 18 Gy) were predictive of poor long-term outcome for cognitive and education ability. In contrast, children who received chemotherapy alone, with or without intrathecal methotrexate, performed similarly to healthy controls. No gender differences were detected for these measures.

**Keywords:** childhood leukaemia; cranial irradiation; intellectual sequelae

Prevention of leukaemia in the central nervous system (CNS) has been a major contributing factor to the increase in survival of children with acute lymphoblastic leukaemia (ALL) so that now 75% of children diagnosed with the disease can be expected to survive (Peckham, 1991). As a result large numbers of childhood ALL sufferers are surviving with the potential to lead normal lives. Thus a major consideration in developing protocols for the treatment of ALL is to limit long-term morbidity without compromising treatment efficiency. This has led us to study the effects of CNS irradiation on cognitive and educational abilities.

Early studies (Soni *et al.*, 1975; Ivnik *et al.*, 1981) of the long-term sequelae of CRT suggested that there were no major neurological or psychological problems associated with the treatment. Since 1980 numerous studies have attempted to evaluate the relationship of preventative treatment for CNS leukaemia and disorders of cognitive functioning (Fletcher *et al.*, 1988; Mulhern *et al.*, 1991). Over time the weight of evidence has indicated that CRT treatment is associated with deficits in intellectual and educational functioning. However, a definitive conclusion is elusive, largely due to numerous methodological flaws inherent in the majority of previous studies. Small sample size, biased sample selection, lack of adequate control samples and inappropriate test protocols lead to difficulties in interpreting and comparing results. Further, lack of recognition of differential effects of various treatment methods, such as dose of CRT, age at treatment and specific treatment protocols, may have added to the current confusion. These difficulties in interpretation are emphasised by the findings of three recent, thorough review articles, which have failed to come to any consensus or conclusion (Williams *et al.*, 1986; Brown *et al.*, 1993; Stehbens *et al.*, 1991).

In order to overcome previous methodological difficulties we studied 100 survivors of ALL and compared them with two groups of controls to allow us to determine the specific effect of CRT on cognitive and educational ability. The two groups of controls used were (1) 50 children who had been treated with chemotherapy but no CRT for a variety of malignancies and (2) 100 healthy controls matched for age, sex and socioeconomic status to the ALL group. The first group was chosen to control for the stress of a potentially fatal disease on the child and his family, for the possible influence of a 'chronic illness' on development and for the possible adverse effects of absence from school and social isolation. They could also serve as controls for the possible toxic effects on the CNS of systemic chemotherapy.

Correspondence: E Smibert, Department of Clinical Haematology and Oncology, Royal Children's Hospital, Parkville, Victoria 3052, Australia
Received 11 April 1995; revised 12 October 1995; accepted 17 October 1995

### Patients and methods

The study investigated three groups of children.

Group 1: 100 children diagnosed with ALL who had been treated with chemotherapy and cranial irradiation.

Group 2: 50 children diagnosed with a variety of malignancies who had received chemotherapy but no cranial irradiation.

Group 3: 100 healthy controls.

Group 1 consisted of children treated for ALL at the Royal Children's Hospital Melbourne, between 1977 and 1987 according to multicentre group Study III, 1977-81 ($n=15$) (Ekert *et al.*, 1980) Study IV, 1981-84 ($n=40$) (Ekert *et al.*, 1990) and ANZCCSG Study V, 1985-92 ($n=45$) (Waters *et al.*, 1992) protocols.

Cranial irradiation was given in children over 2 years of age after remission had been documented following induction chemotherapy. Each child received a course of irradiation of either 24 Gy or 18 Gy given with 4 weekly doses of intrathecal methotrexate as part of prophylaxis against CNS leukaemia. Before cranial irradiation children had received two doses of intrathecal methotrexate given on day 1 and day 22 of induction chemotherapy in Study IV and Study V, but in Study III no preirradiation intrathecal methotrexate was given.

Cranial irradiation (24 Gy) was given in 9 increments over 19 days while 18 Gy irradiation (later protocols) was given in

12 fractions, each of 1.5 Gy over 16–18 days using a megavoltage linear accelerator with two opposed lateral fields. In children younger than 2 years at diagnosis intrathecal methotrexate was given and continued at 8 week intervals until the child reached at least 2 years of age when irradiation was given.

In Study III intrathecal methotrexate was given at 16 week intervals in doses calculated on per $m^2$ basis during continuation therapy for 3 years from the initial documented remission. In Study IV intrathecal methotrexate was given at 16 week intervals in age related doses for 1 year from diagnosis. In Study V those patients who had received craniocervical irradiation did not receive any further intrathecal methotrexate.

In group 2 consisting of children who received chemotherapy and no irradiation, 24 received intrathecal methotrexate and 26 had no intrathecal chemotherapy. The children that received intrathecal methotrexate were treated on the Study V protocol for ALL but did not receive cranial irradiation. They received intrathecal methotrexate in an age-related dose on day 1 and 21 of induction chemotherapy, then weekly for 4 weeks and then once every 8 weeks for 2 years after the first documented remission.

Criteria for inclusion in the two clinical groups were:

(1) treatment to have ceased at least 2 years before cognitive evaluation;

(2) continuous complete remission since the initial diagnosis;

(3) age between 7 and 16 years at time of evaluation to enable administration of a consistent psychometric test protocol across the sample;

(4) no developmental, cognitive or neurological problems before diagnosis.

Children diagnosed and treated at the Royal Children's Hospital that met the above criteria were initially contacted by letter and asked to participate in the study. The first 150 who agreed to participate were included. Four families declined to participate with reasons generally relating to unwillingness on the part of the patient as a result of perceived inconvenience.

Groups 2 and 3 were matched as closely as possible to group 1 with respect to age, sex and socioeconomic status (SES). Group 3 was recruited from schools within the Melbourne metropolitan area. Children enrolled in this group were required to meet selection criteria 3 and 4 above and to have been resident in Australia for at least 5 years to control for ethnicity across samples. Primary and secondary schools within the Melbourne metropolitan area were selected and contacted based on socioeconomic data, with the aim being to achieve a sample representative of the general population. Children within required age groups were selected randomly from class lists and parents were contacted by letter asking for written consent for their child to participate in the study. Table I describes the demographic characteristics of the groups and Table II lists the treatment characteristics of group 1.

### *Methods*

Intellectual ability was measured using the 12 subtests from the Wechsler Intelligence Scale for Children - Revised (WISC-R) (Wechsler, 1974) verbal (VIQ), performance (PIQ) and full-scale (FIQ) intellectual quotients (mean = 100, standard deviation = 15) and scaled scores (mean = 10, standard deviation = 3) for each of the 12 subtests were employed in statistical analyses of intellectual function.

The reading, spelling and arithmetic subtests of the Wide Range Achievement Test - Revised (WRAT-R) (Jastak *et al.*, 1984) were administered to determine the educational abilities of the children, and standard scores (mean = 100, standard deviation = 15) were employed in analyses.

Evaluation took place on a single day over 2 sessions, the order of the administration of the tests being constant. All evaluations were performed on an individual basis by the same psychologist (TG). Groups 1 and 2 were assessed at an outpatient clinic, with TG being blind to group membership. Each child in groups 1 and 2 also underwent general and neurological examination to identify any significant neurological deficits. The testing of healthy controls was performed at the child's school, on an individual basis, also by TG.

### *Statistical analysis*

The three groups were compared using analysis of variance (MANOVA) to determine any differences among the groups with respect to overall IQ, intellectual profile and educational ability. Within group differences were then analysed for group 1 to identify any specific risk factors with respect to demographic and treatment variables.

### Results

### *Comparison across groups*

Using analysis of variance, differences were detected across groups for all summary IQ measures (VIQ, $F(2,247) = 8.98$, $P < 0.001$; PIQ, $F(2,247) = 5.26$, $P < 0.01$; FIQ, $F(2,247) = 8.95$, $P < 0.0001$), with Tukey's post hoc analyses indicating that group 1 achieved significantly lower scores on each of these measures than groups 2 and 3, who performed similarly. Table III provides a summary of these results.

**Table II** Treatment characteristics for group 1

| | *Age at treatment* <br> *<3 years* (n = *28*) | <br> *3–5 years* (n = *42*) | <br> *>5 years* (n = *30*) |
|---|---|---|---|
| Sex | | | |
| males | 15 | 18 | 12 |
| females | 13 | 24 | 18 |
| CRT | | | |
| 24 Gy | 7 | 10 | 3 |
| 18 Gy | 21 | 32 | 27 |
| Neurological signs | | | |
| (no.) | 8 | 0 | 2 |
| ↓co-ordination | 7 | | |
| ↓reflexes | | | 1 |
| nystagmus | 1 | | |
| toe walking | | | 1 |
| Age at testing | | | |
| Mean | 11.3 | 11.7 | 13.3 |
| s.d. | 2.9 | 3.0 | 2.0 |
| Years since treated | | | |
| Mean | 6.0 | 5.2 | 4.0 |
| s.d. | 2.7 | 2.7 | 2.4 |

**Table I** Demographic characteristics of the three groups

| *Group* | n | *No. of males* | *Test age* <br> *Mean* | <br> *s.d.* | *SES*[a] <br> *Mean* | <br> *s.d.* |
|---|---|---|---|---|---|---|
| Group 1 (CRT + Chemotherapy) | 100 | 45 | 12.1 | 2.8 | 4.6 | 1.1 |
| Group 2 (Chemotherapy only) | 50 | 24 | 11.7 | 2.9 | 4.2 | 1.2 |
| Group 3 (Healthy controls) | 100 | 48 | 12.0 | 2.7 | 4.4 | 1.3 |

[a] Daniel's Scale of Occupational Prestige, using fathers' occupation.

**Table III** Intellectual and educational abilities of three comparison groups

| | *Group 1 (CRT+chemo)* | | | *Group 2 (chemo only)* | | | *Group 3 (healthy control)* | | | |
|---|---|---|---|---|---|---|---|---|---|---|
| | *Mean* | *s.d.* | *95% CI for mean* | *Mean* | *s.d.* | *95% CI for mean* | *Mean* | *s.d.* | *95% CI for mean* | F-*values* |
| WRAT-R reading | 88.0 | 18.4 | 84.4–91.7 | 98.1 | 18.9 | 92.7–103.5 | 100.5 | 14.5 | 97.6–103.4 | 14.3** |
| WRAT-R spelling | 87.8 | 16.3 | 84.5–91.0 | 96.8 | 15.7 | 92.3–101.3 | 98.5 | 13.1 | 95.7–101.1 | 14.0** |
| WRAT-R arithmetic | 88.3 | 15.1 | 85.3–91.3 | 98.7 | 14.5 | 94.5–102.9 | 96.2 | 15.6 | 93.1–99.2 | 10.2* |
| WISC-R VIQ | 92.9 | 13.6 | 90.2–95.7 | 100.1 | 13.1 | 96.4–103.9 | 100.2 | 12.5 | 97.7–102.7 | 9.1* |
| WISC-R PIQ | 98.2 | 12.6 | 95.7–100.7 | 104.1 | 13.7 | 100.2–108.0 | 103.1 | 12.1 | 100.7–105.6 | 5.3† |
| WISC-R FIQ | 94.9 | 13.1 | 92.3–97.5 | 102.4 | 13.5 | 98.4–106.1 | 101.7 | 12.9 | 99.3–104.2 | 9.0* |

†$P<0.01$. *$P<0.001$. **$P<0.0001$.

For educational measures similar results were observed. Significant differences were detected on the WRAT-R for reading ($F(2,247)=14.32$, $P<0.0001$), spelling ($F(2,247)=14.00$, $P<0.0001$) and arithmetic ($F(2,247)=10.20$, $P<0.001$). Once again post hoc analyses indicated that group 1 achieved significantly poorer scores than groups 2 and 3, with these latter groups performing similarly. Table III lists mean scores for the groups for each of these educational measures.

Investigation of individual subtest results showed the poorer abilities of group 1, the CRT group, which achieved lowest scores on all 12 WISC-R subtests, with these results reaching statistical significance for 8 of the 12 subsets. In contrast, children treated with chemotherapy alone performed similarly to healthy controls, suggesting no significant effect of chemotherapy on cognitive functioning. There was no correlation between the presence of neurological signs and intellectual or educational deficits. The results have been published and discussed in detail (Anderson *et al.*, 1994).

### *Within group differences*

*Dose of irradiation* Group 1 was subdivided into two groups: (1) those who received 24 Gy CRT ($n=20$) and (2) those who received 18 Gy CRT ($n=80$). These two groups were compared with regard to intellectual and educational abilities, after co-varying for differences in age at which they were treated.

Significant differences were identified between the two groups for PIQ ($F(2,97)=5.18$, $P<0.05$), but not for VIQ ($F(2,97)=3.03$, NS). However, educational measures identified consistent discrepancies between groups with the high-dosage CRT group achieving consistently poorer scores, (reading, $F(2,97)=6.79$, $P<0.01$; spelling, $F(2,97)=4.51$, $P<0.05$; arithmetic, $F(2,97)=11.46$, $P=0.001$) than the group that received 18 Gy CRT. These results are illustrated in Figure 1.

Mean subtest scores for the two groups show that children treated with 24 Gy attain consistently lower scores than those treated with 18 Gy, and also than the healthy controls in all twelve subtests of the WISC-R.

These lower scores represent significant differences between the two dosage groups for the following subtests: information ($F(1,98)=4.06$, $P<0.05$), digit span ($F(1,98)=8.10$, $P<0.01$), picture completion ($F(1,98)=4.39$, $P<0.05$) and vocabulary ($F(1,98)=4,92$, $P<0.05$).

*Age at treatment* Group 1 was subdivided into three groups according to the age at which they received CRT, (i) less than 3 years ($n=28$), (ii) 3–5 years ($n=42$), (iii) greater than 5 years ($n=30$), with the classification based on theoretical knowledge with respect to critical periods of cerebral development. From Figure 2 it can be seen that there was a trend for both groups of children irradiated before 5 years of age to exhibit lower mean IQ scores than those irradiated after 5 years of age. However significant differences were detected only for PIQ ($F(2,97)=4.25$, $P=0.05$). On educational measures similar trends were evident with significant differences across 'age at treatment' groups noted for reading ($F(2,97)=5.13$, $P<0.01$) and arithmetic ($F(2,97)=5.07$, $P<0.01$). In contrast, children receiving CRT after 5 years of age achieved results within the average range and closer to those achieved by the healthy control group.

Mean subtest scores for each of the age at treatment groups were compared. Significant differences were detected among the groups for block design ($F(2,97)=7.82$, $P<0.01$),

**Figure 1** Effect of dose of cranial irradiation on intelligence measured by WISC-R and academic achievement measured by WRAT-R. FIQ, full-scale IQ; PIQ, performance IQ; VIQ, verbal IQ; [hatched box], CRT at 18 Gy ($n=80$); [black box], CRT at 24 Gy ($n=20$). Error bars represent one standard error of the mean.

**Figure 2** Effect of age at which cranial irradiation was administered on intelligence and academic achievement. FIQ, full-scale IQ; PIQ, performance IQ; VIQ, verbal IQ; [black box], CRT aged <3 ($n=28$); [grey box], CRT aged 3–5 ($n=42$); [white box], CRT aged >5 ($n=30$). Error bars represent one standard error of the mean.

information ($F(2,97)=3.75$ $P<0.05$) and coding ($F(2,97)=3.64$ $P=0.05$), with children irradiated at younger ages achieving lowest scores. Similar non-significant trends were observed for all subtest results.

Addressing interaction between age at treatment and dose, it can be seen from Figure 3 that both young age and high dose appear to be risk factors for long-term educational and intellectual functions. No formal statistical evaluation was performed due to the small numbers in the two high-dose groups.

*Gender* Two-way analysis of variance (age at treatment × gender) identified no significant difference in cognitive functions or academic achievement and no interaction effect.

*Intrathecal methotrexate* Group 2 patients that received chemotherapy but no cranial irradiation were subdivided into two groups: those who had received intrathecal methotrexate ($n=24$) and those who had not ($n=26$).

**Figure 3** Academic achievement and intelligence according to age at which cranial irradiation was administered and dose of cranial irradiation. FIQ, full-scale IQ; PIQ, performance IQ; VIQ, verbal IQ; [hatched], CRT <5 at 24 Gy ($n=18$); [hatched], CRT >5 at 24 Gy ($n=3$); [shaded], CRT <5 at 18 Gy ($n=52$); [open], CRT >5 at 18 Gy ($n=27$).

**Figure 4** Effect of intrathecal methotrexate *vs* no intrathecal methotrexate on intelligence and academic achievement in control patients who were treated with chemotherapy but not cranial irradiation. FIQ, full-scale IQ; PIQ, performance IQ; VIQ, verbal IQ; [hatched], IT MTX (no CRT) $n=24$; [solid], no IT MTX (no CRT) $n=26$. Error bars represent one standard error of the mean.

Figure 4 shows that when these two groups are compared with regard to IQ and educational achievement there is no significant difference between the two subgroups on educational measures but that full-scale IQ and performance IQ are significantly different.

### Discussion

The results of this study show that children that receive CRT for the treatment of ALL are adversely affected with regard to their cognitive and educational abilities compared with children that received chemotherapy alone or healthy controls. The mean level of the IQ of group 1 is below the two control groups and the difference is significant. The intellectual problems are generalised but verbal skills are more affected than non-verbal skills. The degree of intellectual deficit is relatively mild, with mean scores for performance IQ, verbal IQ and full-scale IQ within the average range as defined by Wechsler and less than seven IQ points below those achieved by healthy controls. The fact that the IQ levels for the CRT group fall within the average range may explain why some studies have failed to detect any intellectual deficits. With small sample sizes, the power to detect such relatively mild problems would be quite low.

The results with regard to educational ability of the irradiated children are more marked, with their mean scores for reading, spelling and arithmetic being below the average range. Current findings indicate generalised educational deficits rather than the frequently described specific arithmetic deficits (Copeland *et al.*, 1985, 1988; Rourke, 1987).

Social class differences, commonly identified (Trautman *et al.*, 1988) as critical to both intellectual and educational functioning, were controlled for in this study by matching a healthy group of children with the treated group on the basis of the socioeconomic status of the parents. Previous research has also suggested that educational difficulties may be a result of missed schooling (Eiser, 1980; Sawyer *et al.*, 1989) but the observation that children in group 2, who also missed significant school time, are performing similarly to healthy controls argues against this explanation. Thus we have shown that the problem is related to treatment rather than psychosocial factors (Anderson *et al.*, 1994).

### *Dose of irradiation*

A number of previous studies have addressed the impact of high- or low-dose CRT, with little consensus with respect to findings (Trautman *et al.*, 1988; Schuler *et al.*, 1981; Appleton *et al.*, 1990; Mulhern *et al.*, 1992; Halberg *et al.*, 1992; Hirsch *et al.*, 1979). When our group 1 sample was subdivided into those who received a high dose (24 Gy) compared with a lower dose (18 Gy), it was found that the higher dose produced more adverse effects with regard to intellectual and educational abilities. Once again, differences were not large in magnitude, but Figure 1 emphasises the generalised 'dampening' effect of higher doses of CRT, perhaps suggestive of global depression of processing efficiency.

Clinically, cognitive deficits are commonly observed following high-dose CRT (Halberg *et al.*, 1992) and currently most centres use 18 Gy for those children who still receive CRT in their treatment of ALL.

Neurological studies have supported these findings, identifying more significant changes in CNS white matter after high-dose CRT (Brouwers *et al.*, 1990; Price *et al.*, 1975), and our study is consistent with these findings. However, although we found greater adverse effects with high-dose (24 Gy) CRT, children who received 18 Gy CRT did not perform as well as the two groups of controls, and this can be seen by comparing Table 3 and Figure 1. This supports a recent study (Jankovic *et al.*, 1994) showing the negative effects on neurocognitive function using 18 Gy.

As chemotherapy regimens including intrathecal therapy are now generally more intensive than when 12 Gy was used as a radiation dose, it is interesting to speculate whether 12 Gy might be sufficient, particularly for younger children who require cranial irradiation. However, with cranial irradiation only given to 'high-risk' patients now it is unlikely that a study comparing 18 Gy with 12 Gy could be performed as it would take too long to accrue sufficient numbers.

*Age at treatment*

Recently it has been argued that chronological age at the time of CNS insult is a critical factor for outcome, with younger children showing poorer recovery, especially when the insult is of a generalised nature (for example head injury, hydrocephalus) (Rourke, 1987; Ewing-Cobbs *et al*., 1989).

Our study supported this position, showing that children irradiated at a younger age, specifically before the age of 5 years, were adversely affected with regard to intellectual and educational achievement with greater problems occurring for tasks tapping non-verbal/problem solving skills. This is consistent with other studies, emphasising that CRT may be particularly damaging to the young child (Jankovic *et al*., 1994; Jannoun, 1983; Cousens *et al*., 1991). In contrast, the performances of children irradiated after 5 years of age were within the average range.

It is usual to delay CRT in children until they are 2 years old but our results indicate that in order to avoid any adverse effect on cognitive and academic ability CRT should, if at all possible, be delayed until the age of 5.

From Figure 3 it can be seen that a high dose of CRT causes a mild but generalised depression of all cognitive skills in a dose–response type manner, regardless of age at treatment. In contrast, age appears to be more critical to educational skills, with younger age at treatment related to poorer educational skills, irrespective of dosage. Thus, children treated under age 5, who are not yet attending school, have greater educational deficits than those who are already attending school.

This may be due to the fact that children treated under 5 commence school with brain function compromised, and so have difficulty acquiring educational skills. In contrast, children treated after commencing school have some initial educational experience with intact brain function and so may have previously acquired basic educational skills on which they can rely during their schooling.

*Gender*

Gender differences reported in previous studies (Mulhern *et al*., 1991; Jannoun, 1983; Waber *et al*., 1990, 1992: Schlieper *et al*., 1989) were not supported by our data. Our treatment group was randomly selected and perhaps selection criteria for other studies may have biased their results.

*Intrathecal methotrexate*

Intrathecal methotrexate has been documented (Bleyer, 1978) as causing CNS damage in much higher doses than used in our study. However our study shows that in the doses used chemotherapy plus intrathecal methotrexate without cranial irradiation does not affect intellectual or educational ability. However when cranial irradiation is given to children who have received chemotherapy and intrathecal methotraxate, cognitive and academic abilities are adversely affected. It may be that the intrathecal methotrexate renders the brain more susceptible to CRT damage, but this possibility is difficult to investigate as current treatment protocols do not include children receiving CRT without intrathecal methotrexate.

The children who received intrathecal methotrexate had higher WISC-R scores than the controls but their educational abilities as measured by the WRAT-R were the same as those of the controls. Presumably the administration of intrathecal methotrexate does not increase intelligence and therefore children with leukaemia must inherently have an above average intelligence before treatment. This is a perfectly feasible possibility. However, the fact that their educational abilities are similar to the controls suggests that they are not able to achieve their full potential.

Previous research has suggested that there may be a range of psychosocial causes for the intellectual and educational difficulties experienced by children treated with CRT. Emotional and psychosomatic symptoms due to the experience of a potentially fatal illness and school absenteeism have all been implicated as possible causes for lower achievement levels in these children (Trautman *et al*., 1988; Eiser, 1980; Cadman *et al*., 1987; Katz *et al*., 1988). We controlled for these factors in this study by using a group of children who had chemotherapy alone for a variety of malignancies and often longer periods as inpatient using intensive chemotherapy, as for Ewing's tumour or acute myeloid leukaemia. This group performed similarly to healthy controls. The mean age of the three groups was similar so difficulties associated with the acquisition of vital skills, e.g. reading, should have been experienced equally. Other studies have suggested that the parents' level of education is the most important in determining a child's academic achievement. We controlled for this by selecting controls from similar SES groups to the study group.

In conclusion, our findings constitute strong evidence that the difference detected between the children being treated with CRT and non-irradiated children is due to the treatment regimen rather than environmental or social factors. Further, they suggest some specific risk factors with respect to intellectual and educational functions. Firstly, high-dose CRT is a significant risk factor, with this treatment related to a generalised damping of abilities. Secondly, treatment before 5 years of age is also related to poorer educational and intellectual ability. Doses of 18 Gy cranial irradiation in a child older than 5 years does not appear to be detrimental to intellectual and educational ability. Finally, it should be emphasised that the pattern of deficits exhibited by children treated with CRT is suggestive of *mild* impairment in intellectual abilities. However, the nature of these deficits in terms of attentional skills may have particular implications for attainment of educational skills, which are more severely affected. These children may benefit from special assistance to improve their educational outcome. This is important in order to improve the quality of life of survivors of ALL to help them achieve their maximum potential, initially at school and ultimately in the workforce.

**Acknowledgements**
This research was supported by the Royal Children's Hospital Research Foundation, Melbourne, Australia and the Anti-Cancer Council of Victoria, Australia.

### References

ANDERSON V, SMIBERT E, EKERT H AND GODBER T. (1994). Intellectual, educational and behavioural sequelae after cranial irradiation and chemotherapy. *Arch. Dis. Child*, **70**, 476–483.

APPLETON RE, FARRELL K, ZAIDE J AND ROGERS P. (1990). Decline in head growth and cognitive impairment in survivors of acute lymphoblastic leukemia. *Arch. Dis. Child*, **65**, 530–534.

BLEYER WA. (1978). The clinical pharmacology of methotrexate. *Cancer*, **41**, 36–51.

BROUWERS P AND POPLACK D. (1975). Memory and learning sequelae in long-term survivors of acute lymphoblastic leukemia: association with attention deficits. *Am. J. Pediatr. Haematol. Oncol.*, **12**, 174–181.

BROWN RT AND MADAN-SWAIN A. (1993). Cognitive, neuropsychological and academic sequelae in children with leukemia. *J. Learn. Disabil.*, **26**, 74–90.

CADMAN D, BOYLE M, SZATMARI P AND OFFORD DR. (1987). Chronic illness, disability and mental and social well-being: findings of the Ontario Child Health Study. *Pediatrics*, **79**, 805–813.

COPELAND DR, FLETCHER JM, PFEFFERBAUM-LEVINE B, JAFFE N, RIED H AND MAOR M. (1985). Neuropsychological sequelae of childhood cancer in long term survivors. *Pediatrics*, **75**, 745–753.

COPELAND DR, DOWELL RE, FLETCHER JD, BORDEAUX JD, SULLIVAN MP, JAFFE N, FRANKEL LS, RIED HL AND CANGIR A. (1988). Neuropsychological effects of childhood cancer treatment. *J. Child Neurol.*, **3**, 53–62.

COUSENS P, UNGERER JA, CRAWFORD JA AND STEVENS MM. (1991). Cognitive effects of childhood leukemia therapy: a case for four specific deficits. *J. Pediatr. Psychol.*, **16**, 475–488.

EISER C. (1980). Effects of chronic illness on intellectual development. *Arch. Dis. Child*, **55**, 766–770.

EKERT H, WATERS KD, MATTHEWS RN, TAURO GP, RICE MS, SESHADRI R, MAUGER DC, TIERNAN JR, MCWHIRTER WR, O'REGAN P, OLSEN TE AND MATHEWS JD. (1980). A randomized study of intermittent chemotherapy with or without BCG inoculation in maintenance therapy of childhood ALL. *Med. Paediatr. Oncol.*, **8**, 353–360.

EKERT H, WATERS KD, MATTHEWS RN, SMITH PJ, O'REGAN P, RICE M, TOOGOOD I, MAUGER D AND TAURO G. (1990). A randomized study of corticosteroid and non-corticosteroid containing regimes in induction therapy of childhood ALL. *Cancer Therapy and Control*, **1**, 87–95.

EWING-COBBS L, MINER ME, FLETCHER JM AND LEVIN HS. (1989). Intellectual, language and motor sequelae following closed head injury in infants and preschoolers. *J. Pediatr. Psychol.*, **14**, 531–547.

FLETCHER JM AND COPELAND DR. (1988). Neurobehavioural effects of central nervous system prophylactic treatment of cancer in children. *J. Clin. Exp. Neuropsychol.*, **10**, 495–538.

HALBERG FE, KRAMER JH, MOORE IM, WARA WM, MATTHAY KK AND ABLIN AR. (1992). Prophylactic cranial irradiation dose effects on late cognitive function in children treated for acute lymphoblastic leukemia. *Int. J. Radiat. Oncol. Biol. Phys.*, **22**, 13–16.

HIRSCH JF, RENIER D, CZEINICHOW P, BENVENISTE C, PIERRE-KAHN A. (1979). Medulloblastoma in childhood: survival and functional results. *Acta Neurochir.*, **48**, 1–15.

IVNIK RJ, COLLIGAN RC, OBETZ SW AND SMITHSON WA. (1981). Neuropyschological performance among children in remission from acute lymphocytic leukemia. *J. Dev. Behav. Pediatr.*, **2**, 29–34.

JANKOVIC M, BROUWERS P, VALSECCHI MG, VAN VELDHUIZEN A, HUISMAN J, KAMPHUIS R, KINGMA A, MOR W, VAN DONGEN-MELMAN J, FERRONATO L, MANCINI MA, SPINETTA JJ AND MASERA G. (1994). Association of 1800 cGy cranial irradiation with intellectual function in children with acute lymphoblastic leukemia *Lancet*, **344**, 224–227.

JANNOUN L. (1983). Are cognitive and educational development affected by age at which prophylactic therapy is given in acute lymphoblastic leukemia? *Arch. Dis. Child*, **58**, 953–958.

JASTAK JS AND WILKINSON GS. (1984). *The Wide Range Achievement Test-Revised*, Administrative Manual. Jastak Associates: Wilmington, DE.

KATZ ER, RUBINSTEIN CL, HUBERT MC AND BLEW A. (1988). School and social reintegration of children with cancer. *J. Psychosoc. Oncol.*, **6**, 123–140.

MULHERN RK, FAIRCLOUGH D AND OCHS J. (1991). A prospective comparison of neuropsychologic performance of children surviving leukemia who received 18 Gy, or 24 Gy, or no cranial irradiation. *J. Clin. Oncol.*, **9**, 1348–1356.

MULHERN RK, KOVNAR E, LANGSTON J, CARTER M, FAIRCLOUGH D, LEIGH L AND KUN LE. (1992). Long-term survivors of leukemia treated in infancy: factors associated with neuropsychologic status. *J. Clin. Oncol.*, **10**, 1095–1102.

PECKHAM V. (1991). Educational deficits in survivors of childhood cancer. *Paediatrician*, **18**, 147–177.

PRICE RA AND JAMIESON PA. (1975). The central nervous system in childhood leukemia: II. Subacute leukoencephalopathy. *Cancer*, **35**, 306–318.

ROURKE E. (1987). Syndromes of non-verbal learning disability: the fine common pathway of white matter disease/dysfunction. *Clin. Neuropsychologist*, **1**, 209–234.

SAWYER MG, TOOGOOD I, RICE M, HASKELL C AND BAGHURST P. (1989). School performance and psychological adjustment of children treated for leukaemia. *Am. J. Paediatr. Haematol. Oncol.*, **11**, 146–152.

SCHLIEPER AE, ESSELTINE DW AND TARSHIS MA. (1989). Cognitive function in long-term survivors of childhood acute lymphoblastic leukemia. *Paediatr. Haematol. Oncol.*, **6**, 1–9.

SCHULER D, POLCZ A, REVESZ T, KOOS K, BAKOS M AND GAL N. (1981). Psychological late effects of leukemia in children and their prevention. *Budapest Med. and Pediatr. Oncol.*, **9**, 191–194.

SONI SS, MARTEN GW, PITNER SE, DUENAS DA AND POWAZEK M. (1975). Effects of central nervous system irradiation on neuropyschological functioning of children with acute lymphoblastic leukemia. *N. Eng. J. Med.*, **293**, 113–118.

STEHBENS JA, KALEITA TA, NOLL RB, MACLEAN WE, O'BRIEN RT, WASKERWITZ MJ AND HAMMOND GD. (1991). CNS prophylaxis of childhood leukemia: what are the long-term neurological, neuropsychological and behavioural effects? *Neuropsych. Rev.*, **2**, 147–177.

TRAUTMAN PD, ERICKSON C, SHAFFER D, O'CONNOR PA, SITARZ A, CORRERA A AND SCHONFELD IS. (1988). Prediction of intellectual deficits in children with acute lymphoblastic leukemia. *J. Dev. Behav. Paediatr.*, **9**, 122–128.

WABER DP, GIOIA G, PACCIA J, SHERMAN B, DINKLAGE D, SOLLEE N, URION DK, TARBELL NJ AND SALLAN SE. (1990). Sex differences in cognitive processing in children treated with CNS prophylaxis for acute lymphoblastic leukemia. *J. Paediatr. Psychol.*, **15**, 105–122.

WABER DP, TARBELL NJ, KAHN CM, GELBER RD AND SALLAN SE. (1992). The relationship of sex and treatment modality to neuropsychologic outcome in childhood acute lymphoblastic leukemia. *J. Clin. Oncol.*, **10**, 810–817.

WATERS KD. (1992). A randomized clinical trial of modified BFM therapy versus modified high dose asparaginase therapy in childhood acute lymphoblastic leukemia. *Med. Paediatr. Oncol.*, **20**, (abstract 83).

WECHSLER D. (1974). *Manual for the Wechsler Intelligence Scale for Children-Revised.* The Psychological Corp: San Antonio, Tx.